# Optimal Revascularization Strategy on Medina 0,1,0 Left Main Bifurcation Lesions in Type 2 Diabetes

**DOI:** 10.1155/2016/1702454

**Published:** 2016-09-29

**Authors:** Xuwei Zheng, Hongyu Peng, Donghui Zhao, Qin Ma, Kun Fu, Guo Chen, Qian Fan, Jinghua Liu

**Affiliations:** ^1^Department of Cardiology, Beijing Anzhen Hospital, Beijing Institute of Heart, Lung and Blood Vessel Diseases, Capital Medical University, Beijing 100029, China; ^2^Soft Matter and Interdisciplinary Research Center, College of Physics, Chongqing University, Chongqing 401331, China

## Abstract

*Aim*. Diabetes mellitus (DM) is a major risk factor for cardiovascular disease. The implications of a diagnosis of DM are as severe as the diagnosis of coronary artery disease. For many patients with complex coronary artery disease, optimal revascularization strategy selection and optimal medical therapy are equally important. In this study, we compared the hemodynamic results of different stenting techniques for Medina 0,1,0 left main bifurcation lesions.* Methods*. We use idealized left main bifurcation models and computational fluid dynamics analysis to evaluate hemodynamic parameters which are known to affect the risk of restenosis and thrombosis at stented bifurcation. The surface integrals of time-averaged wall shear stress (TAWSS) and oscillatory shear index (OSI) at bifurcation site were quantified.* Results*. Crossover stenting without final kissing balloon angioplasty provided the most favorable hemodynamic results (integrated values of TAWSS = 2.96 × 10^−4^ N, OSI = 4.75 × 10^−6^ m^2^) with bifurcation area subjected to OSI values >0.25, >0.35, and >0.45 calculated as 0.39 mm^2^, 0.06 mm^2^, and 0 mm^2^, respectively.* Conclusion*. Crossover stenting only offers hemodynamic advantages over other stenting techniques for Medina 0,1,0 left main bifurcation lesions and large bifurcation angle is associated with unfavorable flow profiles.

## 1. Introduction

Coronary bifurcation lesion was one of the most challenging subsets in the percutaneous coronary intervention (PCI) due to its lower angiographic success rates and higher risk of procedural complications [1, 2]. Currently, the single stent strategy has been considered the default approach of the treatment for bifurcation lesions [3–5]. However, the optimal stent strategy for Medina 0,1,0 LM bifurcation lesions remains elusive. Precise stent placement and crossover stenting technique have both been proposed to treat these bifurcation lesion subsets. Previous studies showed that stenting from LM to left anterior descending artery (LAD) not only is procedurally feasible but also associated with acceptable clinical outcomes [6–8]. However, some studies found this procedure is sometimes accompanied with significant left circumflex artery (LCX) ostium compromise even if there is no baseline stenosis in the LCX ostium [9, 10]. In many occasions, this may increase complexity of the operation and convert the procedure to a double stenting strategy.

Flow patterns in bifurcations are complex, including vortex formation and creation of zones of low and oscillating wall shear stress which coincide with early intimal thickening. Altered local hemodynamic profile and associated blood flow disturbances caused by the stent implantation are known to be associated with restenosis and thrombosis rates. Our previous work and other computational fluid dynamics (CFD) studies have shown that different stenting techniques bring different hemodynamic conditions and the bifurcation anatomy can be partly influenced by the local flow environment alteration [11–13]. Among geometry factors which may impact on the flow pattern, bifurcation angle has been identified as an important contributor. To the best of our knowledge, the disturbances that various stenting techniques impose on post-PCI coronary flow and the hemodynamic role of bifurcation angle in the Medina 0,1,0 LM bifurcation treatment have not been studied. In the present study, we used a novel method to evaluate hemodynamic conditions and flow patterns at stented LM bifurcations with different bifurcation angles by simulating stenting techniques that are commonly used in clinical practice. The objective of this study was to investigate the hemodynamic effect of bifurcation angle in the Medina 0,1,0 LM bifurcation and provide insights into optimal strategy for this subtype of bifurcation lesions comparing hemodynamic results of different stenting techniques.

## 2. Materials and Methods

### 2.1. Creation of Idealized LM Bifurcation Models

The execution of CFD simulations requires a theoretical vessel model, which is then recreated as a 3-dimensional digital model. Computational models of artery and stents were created in Solidworks 2012, which is a computer aided design package. The bifurcation angle was defined as the angle between the axis of the LAD and the axis of the LCX at its origin. It is well documented that it always falls in between 20° and 120° in the clinical practice and can be divided into acute, right, and obtuse ones. The angle between LM and LAD was defined as absent. The diameter of the LM of the model is 4.0 mm and the diameter of the LCX is 2.5 mm, since usually only side branches with diameters > 2.25 mm are considered for stenting. Three idealized bifurcation models were created with defined bifurcation angle (70°, 90°, and 110°) and Finet's law [14]. The simulated coronary stent closely resembles the strut design and linkage pattern of a third-generation, everolimus-eluting stent (PROMUS Element, Boston Scientific). The cross section of the simulated stent struts was considered square with thickness of 0.081 mm. The dimensions of simulated stents were 16 mm/3.5 mm at the LAD in the precise stent deployment technique and 16 mm/4.0 mm at the LM-LAD in crossover stenting techniques. After placing the solid stent model in the proper position of the bifurcation model, material removal or flex deformation was applied. A Boolean intersection command was finally implemented to subtract the stent from the bifurcation lumen to obtain the final geometry. It was assumed that there was no residual stenosis at the stented vessel after stent implantation, and, in all cases, optimal stent deployment and complete apposition of stent struts against the vessel walls were considered. Residual stenosis at the LCX (Figure 2(c)) was considered an eccentric ostial diameter stenosis due to the combined effect of plaque shift and displacement of the flow divider by the expanded stent struts.

### 2.2. Considered Stenting Techniques

Three single stenting techniques were considered as follows.

#### 2.2.1. Precise Stent Placement

**Figure 1:**
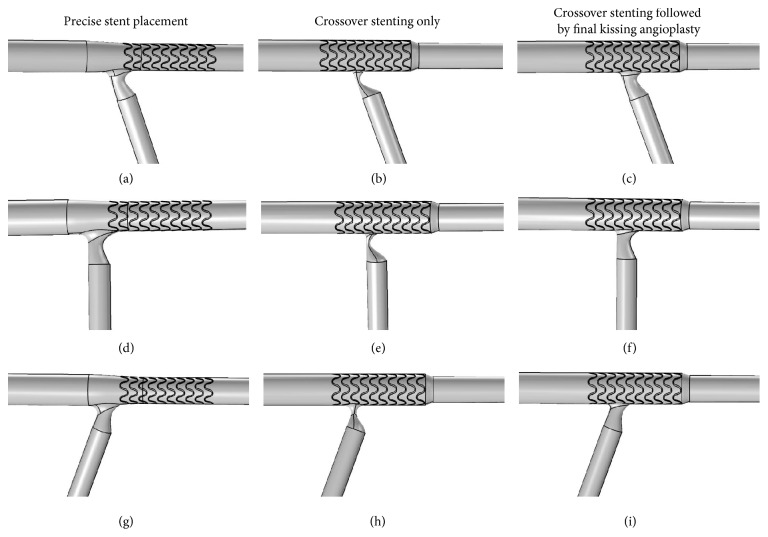
Idealized models of considered stenting techniques under different bifurcation angle conditions. (a), (b), and (c) illustrate stenting techniques under 70° bifurcation angle condition; (d), (e), and (f) under 90° bifurcation angle condition; (g), (h), and (i) under 110° bifurcation angle condition.

In this case, one stent is precisely implanted at the ostial LAD without any intervention at the LCX (Figure 1(a)). It is assumed that the ostial lesion is fully covered by the stent and there is no geographic miss. At the LCX, we considered an eccentric ostial diameter stenosis of 30% due to carina shift.

#### 2.2.2. Crossover Stenting Only

In this case, one stent is implanted from LM to LAD without any intervention at the LCX (Figure 1(b)). Stent implantation causes enlargement of the stented LAD and results in introduction of stent struts inside the bifurcation lumen at the orifice of the LCX. We considered a residual diameter stenosis of 75% due to the combined effect of plaque shift from the LAD and displacement of the carina by the expanded stent struts.

#### 2.2.3. Crossover Stenting Followed by Final Kissing Balloon Angioplasty

In this case of crossover stenting, one stent is implanted from LM to LAD and then kissing balloon inflation is performed so that there are no struts inside the bifurcation lumen (Figure 1(c)). We considered a residual diameter stenosis of 30% because angiographic success is frequently defined as achievement of <50% residual stenosis by any percutaneous method.

Defined boundary conditions are imposed and the Navier-Stokes equations that describe the laminar motion of fluids are numerically solved using numeric grids. The simulations were conducted using the commercial software COMSOL Multiphysics (version 4.2). The following assumptions were made.

**Figure 2:**
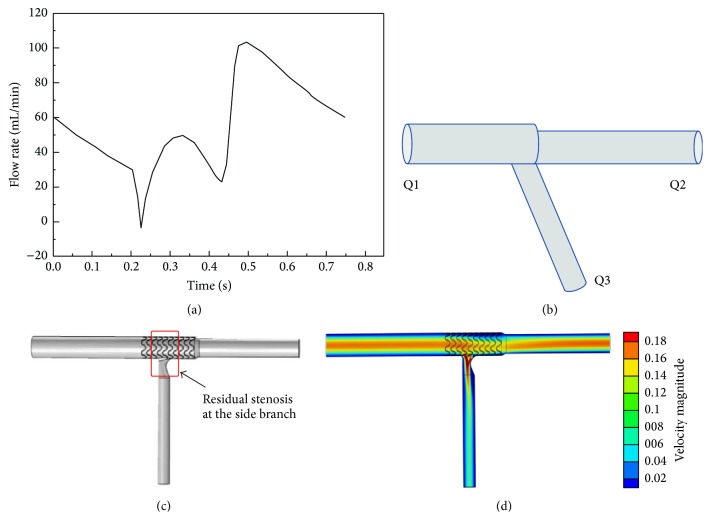
(a) Imposed inflow boundary conditions. (b) Outflow boundary conditions. (c) Idealized left main bifurcation model of crossover stenting followed by final kissing balloon angioplasty under right bifurcation angle condition. A residual diameter stenosis of 30% is considered at the proximal LCX. (d) The numeric solution of flow velocity distribution.

The blood density is set as 1.06 × 10^3^ kg/m^3^ and its viscosity is set as 3.5 × 10^−3^ Pa·s. The artery walls are assumed to be rigid and no deformation is taken into account. The blood is considered as a Newtonian incompressible fluid with flow based on human left coronary artery pulsatile velocity measurements applied at the inlet of the vessel (Figure 2(a)). For the outlets, the downstream microcirculation resistance was considered and Murray's law was used to estimate the boundary conditions (Figure 2(b)).

The hemodynamic parameters that were assessed at stented coronary bifurcations through CFD simulations were the time-averaged wall shear stress (TAWSS) and the oscillatory shear index (OSI). Consider

(1) TAWSS=1T∫0TWSSs,tdt,OSI=121−1/T∫0TWSSs,tdt1/T∫0TWSSs,tdt,

where *T* is the overall interval of the cardiac cycle and *s* is the position on the vessel wall.

TAWSS accounts for the frictional force per unit area that is exerted by the flowing blood to the vascular wall. It was calculated by integrating each nodal WSS magnitude over the cardiac cycle. Previous studies have demonstrated that lower TAWSS values (lower than 0.4 Pa) are associated with cellular proliferation, intimal thickening, and inflammation [15]. OSI is a measure of the extent of oscillatory flow behavior. It is used to identify regions on the vessel wall subjected to highly oscillating WSS values during the cardiac cycle. Low OSI values (close to 0) occur at sites where flow disruption is minimal, whereas high OSI values (close to 0.5) indicate that WSS vector is subject to large variations, and WSS can be very low or easy to change direction, which means in that case flow is stopped or reversed. The oscillatory shear and associated reversed flow and recirculation zones have been found to promote endothelial dysfunction and intimal hyperplasia [16]. Studies have shown that there is a putative link between hemodynamic disturbances and the risk of restenosis and thrombosis [17, 18]. It is plausible that the risk of complications would be higher if regions of the bifurcation are continuously exposed to unfavorable flow profiles, that is, low TAWSS and high OSI. In the present study, three single stenting techniques commonly used in Medina 0,1,0 bifurcation lesions PCI were comparatively evaluated in terms of the induced flow pattern alteration at the subregion of bifurcation site.

## 3. Results

**Figure 3:**
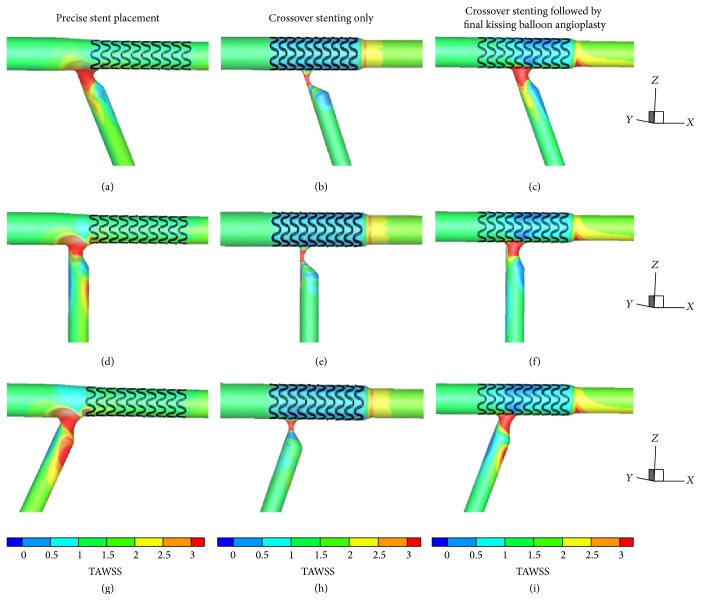
Time-averaged wall shear stress (TAWSS) distribution at the bifurcation for the considered three stenting techniques under different bifurcation angle conditions. (a), (b), and (c) illustrate stenting techniques under 70° bifurcation angle condition; (d), (e), and (f) under 90° bifurcation angle condition; (g), (h), and (i) under 110° bifurcation angle condition.

**Figure 4:**
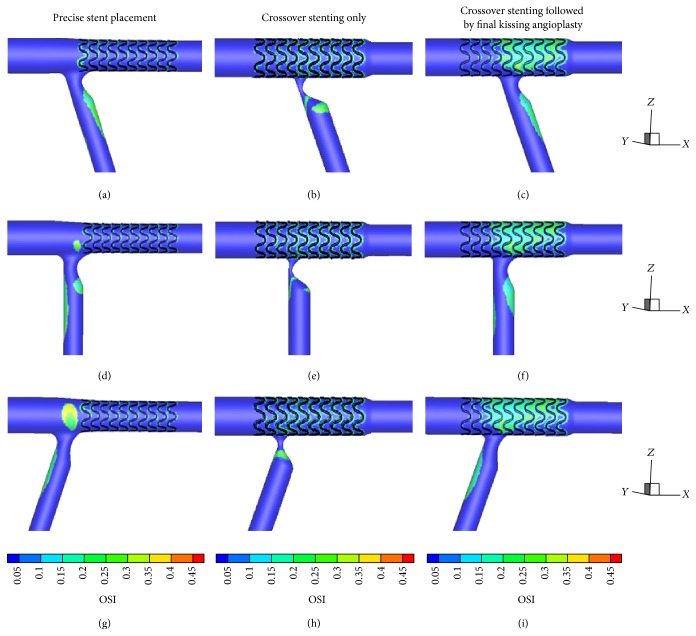
Oscillatory shear index (OSI) distribution at the bifurcation for the considered three stenting techniques under different bifurcation angle conditions. (a), (b), and (c) illustrate stenting techniques under 70° bifurcation angle condition; (d), (e), and (f) under 90° bifurcation angle condition; (g), (h), and (i) under 110° bifurcation angle condition.

Figures 3 and 4 give the TAWSS and OSI distributions for the three considered stenting techniques under different bifurcation angle conditions, that is, acute, right, and obtuse angles.

### 3.1. Stenting Technique under Different Bifurcation Angles

#### 3.1.1. Precise Stent Placement

Low WSS regions are mainly located at the proximal LCX and gradually transferred from lateral wall to medial wall from 70° to 110° (Figures 3(a), 3(d), and 3(g)). High WSS regions are identical under different bifurcation angles and mainly located at the flow divider and the medial wall of distal LM. In comparison with the conditions of 70° and 90°, low WSS region at the LM-LAD junction enlarged significantly when bifurcation angle is 110° (Figure 3(g)).

#### 3.1.2. Crossover Stenting Techniques

The bifurcation angle change does not impose any significant flow alterations on the main branch. At the stented region, the distributions of WSS and OSI are identical for the two crossover stenting techniques both proximally and distally to the flow divider: proximally there is a region of low WSS and high OSI at the lateral wall of distal LM and distally there is a region of elevated WSS at the edge between the stented and nonstented vessel due to the tapering vessel that causes flow acceleration. Flow conditions differentiate among cases at the LCX: accelerated flow caused by the residual stenosis results in high flow velocity and WSS value at the proximal LCX. In the cases of crossover stenting followed by final kissing balloon angioplasty, low WSS area is gradually transferred from lateral wall to medial wall with the alteration of bifurcation angle (Figures 3(c), 3(f), and 3(i)). These phenomena are less distinct in the cases of crossover stenting only.

### 3.2. Stenting Techniques under the Same Bifurcation Angle

Low WSS area of three stenting techniques is consistently located at the lateral wall of the stented region, as well as opposite to flow divider in the LAD and LCX in the cases of 70°. Except the whole stenotic region, high WSS area can be observed at the medial wall of distal LM in the case of precise stent placement (Figure 3(a)) and at the stent edge of LAD in the case of two crossover stenting techniques. Different from the WSS distribution in the cases of 70°, a new low WSS and elevated OSI area can be seen at the proximal LAD in 90° and 110° which are unique phenomena in the cases of precise stent placement (Figures 3(d), 3(g), 4(d), and 4(g)). This is probably owing to intensifying flow perturbations and reverse flow formation at the carina in the cases of larger bifurcation angles.

### 3.3. Comparison of Techniques with Different Bifurcation Angles

When comparison of techniques under different bifurcation angle conditions is considered, the surface integrals of TAWSS and OSI were calculated at a subregion of bifurcation site which was fixed in Figure 2(c). The integral of each index was normalized to that of the stenting technique that provided the most hemodynamically favorable results, that is, highest TAWSS (Table 1) and lowest OSI (Table 2). The ranking of the stenting techniques in Table 1 follows a descending order in the case of each defined bifurcation angle. From Table 1, we can derive that crossover stenting only gives the optimal results whatever the bifurcation angle is. The flow patterns of 70° are more favorable in the case of two crossover stenting techniques when compared with other bifurcation angles. However, in the cases of precise stent placement, the optimum result is found under the bifurcation angle of 70° and is comparable to crossover stenting only. More disturbed flow can be observed in these cases with the angle going larger. Additionally, we calculated the total area of the bifurcation region that is subjected to OSI values greater than specific predefined thresholds for each stenting technique. The results, shown in Table 3, indicate that crossover stenting only approach under acute bifurcation angle condition results in smaller arterials segments at which flow is stopped or reversed.

## 4. Discussion

Although several studies have demonstrated that single stent strategy is superior to systematic double stent techniques in true bifurcation lesions, the optimal single stenting technique for Medina 0,1,0 LM bifurcation lesions is still under debate [3, 4, 19]. We have made several novel observations in our present experiment. First, we have compared stenting techniques on these bifurcation lesion subsets and have demonstrated for the first time in an idealized model that crossover stenting only but not precise stent placement gives the optimal overall hemodynamic results. Second, we have demonstrated that kissing appeared to be hemodynamically detrimental after crossover stenting in these lesion subsets. Third, we have provided evidence that bifurcation angle is an important element to be considered before approaching a bifurcation lesion. Precise stent placement might also be an acceptable choice under acute angle conditions. However, crossover stenting techniques should be preferred when there is right or obtuse bifurcation angle.

Crossover stenting technique seems to be the quickest and easiest method to allow for safe full coverage of the LAD ostium. The major concerns for this simple technique are stent malapposition in the distal LM and LCX jailing. Optimal stent deployment and complete apposition of stent struts against the vessel wall were supposed in the current study. Regarding the LCX compromise, some studies showed significant ostial side branch stenosis after crossover stenting technique may cause persistent ischemia and symptoms [20, 21]. The mechanism of side branch jailing during bifurcation stenting includes plaque shift from main vessel to side branch, carina shift toward the side branch lumen, the presence of stent struts covering the ostium, and coronary vasospasm [9, 21]. However, a recent study by Kang et al. has demonstrated that the use of single stent technique rarely resulted in the functional LCX compromise in LM bifurcation lesions with mild LCX ostial disease [22]. Our computational findings are in keeping with the results because, in our considered models, although we simulated a relative tight residual stenosis in the LCX ostium after crossover technique, the distal LCX flow was not significantly affected. Regarding whether the final kissing balloon inflation should be performed after crossover stenting, our findings are in keeping with the results of clinical trials that documented that kissing inflation can be avoided in bifurcation lesions uneventfully treated with crossover technique [23, 24]. Recent studies have demonstrated that a stent-induced increase in lumen diameter of the distal MV forces the position of the carina into the SB ostium and this carina shift is the main mechanism of the SB narrowing after MV stent implantation [25, 26]. Bifurcation lumen changes after crossover stent implantation are determined primarily by conformational changes in vessel geometry [27]. A recent IVUS study after stent implantation from the LMCA to the LAD demonstrated that most of lumen losses of the LCX are due to carina shift, and kissing balloon technique can adjust carina shift but cannot improve plaque shift [28]. However, functional LCX stenosis is poorly predicted by a small minimal lumen area. Our quantification revealed that the total area of low TAWSS was essentially the same after crossover stenting and after kissing inflation. While kissing inflation restored carina position, it also caused low TAWSS and high OSI in the distal main vessel due to slight repositioning of the carina. Thus, although postkissing inflation provides an excellent result in terms of patency, from a fluid dynamics perspective there are only modest differences, indicating that the potential for neointimal hyperplasia or thrombus formation may be unchanged.

Even though using multiple angiographic views and large volumes of contrast to assist in stent implantation is sometimes time-consuming and frustrating, precise stent placement remains a common approach used for ostial lesion stent implantation in clinical practice. However, recently, several studies showed that conventional visual estimates with angiographic assistance may not be reliable. In a retrospective study, Dishmon et al. found that angiographically guided stenting leads to a high incidence of proximal and distal stent misplacement [29]. The inaccurate stent positioning resulted in significantly higher target lesion revascularization rates when compared to patients without geographic miss. Nowadays, some studies report that the use of a nitinol-based stent-positioning tool (the “Ostial Pro”) appeared to be promising, but the long-term outcomes have not yet been reported [30]. Although models in our study assume accurate stent placement without geographic miss, it was found that it still resulted in lower WSS and high OSI when compared to crossover stenting only technique. We can speculate that the high WSS on the medial wall of distal LM facilitates the local platelet activation and other thrombogenic factors accumulation. And this effect may finally intensify the downstream disturbed flow and vortex formation caused by low WSS at the LM-LAD junction. Despite the fact that we cannot directly link hemodynamic disturbances and the risk of restenosis and stent thrombosis, it is plausible that the risk of restenosis would be higher if the subregions of bifurcation sites are continuously exposed to unfavorable hemodynamic conditions. However, in our simulations, precise stent placement is still an acceptable approach under acute bifurcation angle conditions. Especially in some cases where the diameter of LM is much larger than LAD, precise stent placement should be considered first because the mismatch can lead to stent malapposition in the distal LM.

The hemodynamic performances of stenting techniques with different bifurcation angles were also studied in our simulation. Our findings are in keeping with the clinical results that documented that large bifurcation angle is associated with a greater risk of restenosis rates [31, 32]. It was found that, in all simulated single techniques, lower WSS and elevated OSI can be observed under large bifurcation angle conditions. We can observe that there are apparent areas of high WSS adjacent to those of low WSS under larger bifurcation angle conditions. The high WSS can possibly stimulate the local platelet activation and aggregation. Subsequently, the flow stagnation and neointimal hyperplasia created by low WSS downstream may be greatly intensified by the former. This may partly explain the underlying reasons for high bifurcation angles associating worse outcomes.

## 5. Study Limitations

Several limitations of the current study should be acknowledged. First, the considered models represent idealized Medina 0,1,0 LM bifurcation lesions. Angle between LM and LAD is not taken into account and the possible size mismatch between LM and LAD is not concerned in the present study. This may increase the possibility of stent malapposition in the distal LM for crossover stenting techniques. Several studies have shown that the incomplete stent apposition is increasingly recognized as a hallmark risk for restenosis and late stent thrombosis [33, 34], while complete apposition of stent struts and optimal stent implantation were assumed in our study. Second, the simulated residual stenosis of LCX was considered the same under different bifurcation angles and relatively tight for bifurcations with mild stenosis in the LCX ostium, although in real life the extent of side branch compromise could possibly be affected by the bifurcation angle. Third, the modification of bifurcation angles after stent deployment was not concerned, although it can be observed in some clinical studies [35]. At last, our study deals with immediate hemodynamic results after stent deployment. The long-term clinical outcomes of stenting techniques cannot be deduced from our study.

## 6. Conclusions

In our simulations, crossover stenting only offers hemodynamic advantages over other stenting techniques for Medina 0,1,0 left main bifurcation lesions and large bifurcation angle is associated with unfavorable flow profiles. Randomized controlled clinical trials are needed to confirm the findings of this observational study.

## Figures and Tables

**Table 1:** Surface integrals of TAWSS for considered stenting techniques under different bifurcation angle conditions.

	TAWSS, ×10^−4^ N	Normalized TAWSS
Bifurcation angle = 70°		
Crossover stenting only	2.96	1.00
Crossover stenting followed by final kissing balloon angioplasty	2.02	0.68
Precise stent placement	2.78	0.94
Bifurcation angle = 90°		
Crossover stenting only	2.35	0.79
Crossover stenting followed by final kissing balloon angioplasty	1.91	0.65
Precise stent placement	1.84	0.62
Bifurcation angle = 110°		
Crossover stenting only	2.27	0.77
Crossover stenting followed by final kissing balloon angioplasty	1.85	0.63
Precise stent placement	1.32	0.45

TAWSS, time-averaged wall shear stress; the calculated subregion of bifurcation sites is described in Figure 2(c).

**Table 2:** Surface integrals of OSI for 3 considered stenting techniques under different bifurcation angle conditions.

	OSI, ×10^−6^ m^2^	Normalized OSI
Bifurcation angle = 70°		
Crossover stenting only	4.75	1.00
Crossover stenting followed by final kissing balloon angioplasty	4.88	1.03
Precise stent placement	5.06	1.07
Bifurcation angle = 90°		
Crossover stenting only	5.11	1.08
Crossover stenting followed by final kissing balloon angioplasty	5.74	1.21
Precise stent placement	6.32	1.33
Bifurcation angle = 110°		
Crossover stenting only	5.43	1.14
Crossover stenting followed by final kissing balloon angioplasty	6.08	1.28
Precise stent placement	7.27	1.53

OSI, oscillatory shear index; the calculated subregion of bifurcation sites is described in Figure 2(c).

**Table 3:** Bifurcation area subjected to OSI specific thresholds for considered stenting techniques under different bifurcation angle conditions.

	Bifurcation areas, mm^2^		
	OSI > 0.25	OSI > 0.35	OSI > 0.45
Bifurcation angle = 70°			
Crossover stenting only	0.39	0.06	0.00
Crossover stenting followed by final kissing balloon angioplasty	0.51	0.09	0.00
Precise stent placement	0.38	0.07	0.00
Bifurcation angle = 90°			
Crossover stenting only	0.42	0.07	0.00
Crossover stenting followed by final kissing balloon angioplasty	0.63	0.11	0.01
Precise stent placement	0.44	0.10	0.00
Bifurcation angle = 110°			
Crossover stenting only	0.43	0.05	0.00
Crossover stenting followed by final kissing balloon angioplasty	0.65	0.11	0.02
Precise stent placement	0.46	0.12	0.04

OSI, oscillatory shear index.
